# Computerized Tomography-Based Volumetric Analysis of The Maxillary, Sphenoid, and Frontal Sinuses: An Observational Study

**DOI:** 10.31729/jnma.8859

**Published:** 2025-01-31

**Authors:** Ashish Dhakal, Bikash Lal Shrestha, Monika Pokharel, Samip Shrestha, Subindra Karki, Krishna Sundar Shrestha

**Affiliations:** 1 Department of ENT-HNS, Dhulikhel Hospital, Kathmandu University Hospital, Kavre, Nepal; 2 Department of Radiology, Dhulikhel Hospital, Kathmandu University Hospital, Kavre, Nepal

**Keywords:** *frontal sinus*, *maxillary sinus*, *paranasal sinuses*, *sphenoid sinus*, *volumetric analysis*

## Abstract

**Introduction:**

Volumetric analysis of the paranasal sinuses can help evaluate disease extent, treatment, and sinus surgery outcome. This study aims to determine the baseline volumes of frontal, maxillary, and sphenoid sinuses.

**Methods:**

An observational cross-sectional study was conducted in a tertiary care center after obtaining ethical approval (Reference number: 264/2021). Computerized Tomography Scan head or Computerized Tomography Scan nose/Paranasal sinus images of patients across all age groups from January to December 2022 were analyzed. Sinus volumes were calculated using Digital Imaging and Communications in Medicine software.

**Results:**

The study included 154 patients, median age was 29 years (IQR:22.75 - 39.25) and 84 (54.5%) were male. The median volume of the maxillary sinus was 14.30 cm^3^ (IQR:10.76 - 18.02), with the right side at 13.82 cm^3^ (IQR:10.34 - 17.39) and the left at 14.62 cm^3^ (IQR:11.11 - 18.75). The median volume of the sphenoid sinus was 4.52 cm^3^ (IQR:3.02 - 6.03), with the right side at 4.42 cm^3^ (IQR:2.61 - 6.84) and the left at 3.95 cm^3^ (IQR:2.16 - 6.47). The median volume of the frontal sinus was 2.59 cm^3^ (IQR:1.42 - 4.59), with the right side at 2.27 cm^3^ (IQR:0.94 - 4.12) and the left at 3.04 cm^3^ (IQR:1.22 - 5.04).

**Conclusions:**

Male population had larger volumes across all types. The maxillary and frontal sinuses had the largest and smallest volumes respectively.

## INTRODUCTION

Sinuses are air-filled cavities in the skull that play a crucial role in physiological functions like air temperature and humidity regulation, voice resonance, and protection of cranial structures.^1-3^ Computerized Tomography (CT) scans have become essential for diagnosing and managing sinus disease, providing detailed information about anatomical features, and guiding treatment decisions.^4-6^ CT-based volumetric assessments have surpassed plain radiography and cadaveric studies, with some studies using three-dimensional (3D) models for more accurate estimation.^4,7-10^

In the context of modern CT scanning techniques, knowing the normal volume of sinuses can help the doctor identify the pathologic development of structures with relevant clinical correlation.^11^ Knowledge of baseline volumetric status in our population is not known. This study aims to provide baseline volumetric data of the maxillary, sphenoid, and frontal sinuses using CT imaging, essential for planning sinus surgeries, diagnosing diseases, and enhancing clinical outcomes. The findings can bridge a critical knowledge gap and serve as a valuable reference for Ear Nose and Throat (ENT) surgeons, radiologists, and researchers in Nepal.

## METHODS

This observational cross-sectional study was conducted in the Department of Otorhinolaryngology and Head & Neck Surgery at the Kathmandu University Dhulikhel Hospital, a tertiary care center located in Dhulikhel, Kavre. The study was approved by the Institutional Review Committee (Reference number: 264/2021). This study includes CT images of all age group patients who had undergone CT head or CT nose/Paranaal sinus (PNS) from January to December 2022. However, CT images of patients with a history of nose and paranasal sinus surgery, a history of maxillofacial trauma, a history of sinonasal disease, congenital anomaly of nose and PNS and malignancy, and CT scans with poor technical quality were excluded from the study.

The CT scan analysis was done in a 128-slice Siemens somatom perspective machine. Patients were positioned in the supine position, and using the parameters 130kV, 145 mAs, and a scan time of 3.5 seconds, a volumetric axial CT scan was taken with 3mm slices from the frontal sinus to the floor of the maxillary sinus. Multiplanar reconstruction was done using 1mm thin slices with 0.5mm intervals, and images were obtained in all planes. For visualization and delineation, the axial section was selected and complemented sagittally and coronally. The volume of the specific sinus was measured using open-source DICOM imaging software InVesalius® 3.1.1 (CTI, Renato Archer Information Technology Center, Brazil).

After completion of the marking process, a 3D image and volume rendering were done by using the software. The volume of the individual sinus was calculated in cubic centimeters. All the observations and calculations were made by a single observer and all measurements were then approved by the expert radiologist observer.

Data entry and analysis were done using SPSS Statistics for Windows, version 21.0 (SPSS Inc., Chicago, III., USA). Frequency and percentages were used to represent categorical data, and continuous data was expressed in terms of median and interquartile range (IQR).

## RESULTS

A total of 154 patients were enrolled in the study with a median age of 29 (IQR:22.75 - 39.25) years with age ranging between 4-91 years. There were a total of 84 (54.5%) male and 70 (45.5%) female in the study.

The median volume of the maxillary sinus on both sides was 14.30 (IQR:10.76 - 18.02)cm^3^. The median volume of the right maxillary sinus was found to be 13.82 (IQR:10.34 - 17.39) cm^3^ and that of the left side was 14.62 (IQR:11.11 - 18.75) cm^3^. In the male population, the median volume of the right maxillary sinus was 15.68 (IQR:10.49 - 19.03) cm^3^ and the left maxillary sinus was 15.61 (IQR:12.56 - 20.44) cm^3^ whereas, in the female, the median volume of the right maxillary sinus was 13.40 (IQR:9.99 - 15.72) cm^3^ while the left maxillary sinus was 12.63 (IQR:9.81 - 17.40) cm^3^.

The median volume of the sphenoid sinus on both sides was 4.52 (IQR:3.02 - 6.03) cm^3^. The median volume of the right sphenoid sinus was found to be 4.42 (IQR:2.61 - 6.84) cm^3^ and the left sphenoid side was 3.95 (IQR:2.16 - 6.47) cm^3^. In the male population, the median volume of the right sphenoid sinus was 4.50 (IQR:2.64 - 7.05) cm^3^ and the left sphenoid sinus was 4.39 (IQR:2.21 - 7.67) cm^3^ whereas, in the female population, the median volume of the right sphenoid sinus was 4.32 (IQR:2.49 - 6.01) cm^3^ while the left sphenoid sinus was 3.43 (IQR:2.04 - 5.35) cm^3^.

The median volume of the frontal sinus on both sides was 2.59 (IQR:1.42 - 4.59). The median volume of the right frontal sinus was found to be 2.27 (IQR:0.94 - 4.12) cm^3^ and the left frontal sinus was 3.04 (IQR:1.22 - 5.04) cm^3^. In the male population, the volume of the right frontal sinus was 2.80 (IQR:1.23 - 5.53) cm^3^ and the left frontal sinus was 3.83 (IQR:1.96 - 6.06) cm^3^ whereas in the female population, the median volume of the right frontal sinus was 1.87 (IQR:0.67 - 2.97) cm^3^ while the left frontal sinus was 2.34 (IQR:0.93 - 4.15) cm^3^.

## DISCUSSION

The anatomy of the paranasal sinus varies from person to person and is very complex. This study analyzed paranasal sinus volume of the maxillary, sphenoid, and frontal sinuses in 154 patients (median age 29 years; IQR 22.75 - 39.25). This study offers crucial insights into the anatomical variations that exist between genders and the comparative sizes of the sinuses. The maxillary sinuses were the largest, with median volumes of 13.82 cm^3^ (right) and 14.62 cm^3^ (left), while the frontal sinuses were the smallest at 2.27 cm^3^ (right) and 3.04 cm^3^ (left). Males consistently had larger sinus volumes than females across all sinus types, showing notable gender differences and asymmetry. These findings have potential clinical and surgical relevance.

Among the sinuses, the maxillary sinuses were the largest, with median volumes of 13.82 cm^3^ (IQR:10.34 - 17.39) on the right and 14.62 cm^3^ (IQR:11.11 - 18.75) on the left. In our study, the findings indicate a consistent pattern where the left maxillary sinus is slightly larger than the right in both males and females. This observation aligns with previous anatomical studies that have suggested a tendency for asymmetry in sinus development, which could have significant clinical implications, particularly in the context of sinus-related pathologies such as sinusitis. ^1,10-13^ This slight predominance of the left maxillary sinus is noteworthy, as it may influence clinical presentations of sinus conditions. For instance, the drainage pathways and the potential for infection may be affected by this anatomical variation.

The gender-specific findings reveal that males exhibit larger maxillary sinus volumes compared to females, with median values of 15.68 cm^3^ (IQR:10.49 - 19.03) for the right and 15.61 cm^3^ (IQR:12.56 - 20.44) for the left in males, in comparison women had median values of 13.40 cm^3^ (IQR:9.99 - 15.72) and 12.63 cm^3^ (IQR:9.81 - 17.40) on the right and left, respectively. These results are also consistent with some previous studies.^7,12,14^ This difference in volume may be linked to various factors, including overall craniofacial morphology, hormonal influences, or genetic predispositions.^10^ These findings highlight the necessity for further investigation into how these factors contribute to sinus development and disease susceptibility.

The volumetric analysis of the sphenoid sinuses is similar to the findings of Oliviera et al and Tuang et al., with the right sinus measuring an average of 4.86 cm^3^ and the left 4.57 cm3.^5,15^ The sphenoid sinuses, while smaller, also showed gender differences. Their median volumes were 4.42 cm^3^ (IQR:2.61 - 6.06) on the right and 3.95 cm^3^ (IQR:2.16 - 6.47) on the left, with men again displaying larger measurements. The sphenoid sinuses, located centrally within the skull, may exhibit less variability than the maxillary sinuses due to their anatomical positioning and developmental patterns.^5^ This stability could suggest a more uniform growth process, which may be less influenced by external factors compared to the maxillary sinuses.

The frontal sinuses showed the most asymmetry and had the smallest volumes overall, with a median volume of 2.27 cm^3^ (IQR:0.94 - 4.12) for the right and 3.04 cm^3^ (IQR:1.22 - 5.04) for the left. Male participants had larger frontal sinuses, with medians of 2.80 cm^3^ (IQR:1.23 - 5.53) and 3.83 cm^3^ (IQR:1.96 - 6.96) on the right and left, respectively. The pronounced difference in frontal sinus volume could reflect developmental factors, including the influence of testosterone during puberty, which may affect craniofacial growth patterns. ^10,11^ The anatomical positioning of the frontal sinuses may also play a role in their susceptibility to conditions like frontal sinusitis, which can be exacerbated by variations in size and drainage pathways.^2,10^

The developmental timeline of these sinuses further elucidates the observed volumetric differences. The frontal sinus begins its development during the fourth month of gestation as an upward extension of the anterosuperior ethmoidal cells in an area termed as the frontal recess.^11^ By the age of six, the frontal sinus can typically be demonstrated radiographically, indicating that its development is relatively early in the life span.^11,12^ In contrast, the maxillary sinus presents as a small sac at birth, with two significant periods of pneumatization occurring between birth and two years of age and again between 7 to 12 years of age. This slow development continues until the late teenage years between 14 to 18 years of age, suggesting that the maxillary sinuses may be more susceptible to environmental factors during their growth phases.^11,16^ Ikeda reported that the maxillary sinus reaches adult dimensions at 12-15 years of age, and this value is maintained until 20 years of age.^17^

The sphenoid sinus pneumatization begins at age 2-3 years and reaches its adult dimensions by 12-14 years of age, which is consistent with the findings of this study regarding its relative stability in size.^16^ Barghouth et al. reported that there were no significant differences in the volume of the maxillary sinus between the left and right sides when considering all ages, which contrasts with the findings in this study.^10^ This discrepancy could be attributed to variations in sample populations or methodologies used in different studies.

A limited sample size may not adequately represent the diverse ethnic groups and anatomical variations according to different age groups within the Nepalese population. Also, the study was limited to a single center with a limited data set which can limit the generalizability of our findings. Also, the lack of universally accepted protocols for volumetric measurement may lead to inconsistencies in the methodology or difficulty comparing results with studies from other populations. Broader inclusion with clusters of multiple centers involving individuals of various ethnic and regional populations needs to be included for better generalization.

## CONCLUSIONS

Maximum volume was that of the maxillary sinuses, and volumes of all types of sinuses were larger in males. The frontal sinuses were the smallest and most asymmetric.
